# The effects of landscape on visual preference and fatigue recovery among university students: Differences in gender, grade level and major

**DOI:** 10.1371/journal.pone.0330694

**Published:** 2025-08-21

**Authors:** Chenyu Zheng, Ming Fang, Yue Zhang, Xinyu Liu, Zhihuan Huang

**Affiliations:** College of Architecture and Design, University of South China, Hengyang, China; Universita degli Studi di Roma Tor Vergata, ITALY

## Abstract

Exposure to natural landscapes has been shown to affect both physiological and psychological well-being, with the extent of these effects varying across different landscape types. However, the underlying mechanisms remain poorly understood. The association among stress reduction, environments characteristics and individual differences requires further investigation, particularly considering the complexity of landscape attributes and the variability of personal responses. In this study, 98 university students participated in a survey to evaluate the effects of different landscape types on visual preference and fatigue recovery. Physiological data (blood pressure, heart rate), psychological data (Perceived Restorative Scale), and visual preferences were analyzed before and after participants viewed the images of eight representative landscape space types: mountain, field, waterscape, lawn, desert, forest, artificial nature, plant. The results indicated that landscape type significantly influenced both physiological responses and emotional states, as well as participants’ perceived recovery from stress. Among the eight landscape spaces, water features and forests were reported to be the most restorative. Compared to freshmen, juniors exhibited greater improvements in physical and psychological recovery, alongside more positive evaluations of the environments. Notably, the desert landscape elicited varied responses depending on participants’ grade level and gender, suggesting that restoration effects may be modulated by individual characteristics. This may reflect an evolutionary predisposition to prefer natural features that enhance survival. These findings contribute to environmental psychology and provide valuable insights for educational practice and environmental design.

## 1. Introduction

Rapid urbanization has led to a growing population in cities. However, urban environments pose substantial challenges to physical and mental health, including increased exposure to noise [1], air pollution [2], and chronic stress [3,4], all of which have been associated with an increased risk of mental health disorders [5,6]. As a densely populated area in the city, university campuses may expose students to a high level of environmental stress. The sources of stress for students are typically attributed to academic workload, financial pressures, time management difficulties, health concerns, and the pursuit of personal development, all of which have become widespread global issues [7,8]. A survey from the WHO Mental Health Surveillance System showed that the global clinical anxiety detection rate among students aged 18–25 rose from 21.4% in 2015 to 34.7% in 2021 [9], with this increasing trend being particularly notable in East Asia. Similarly, the Chinese Center for Disease Control and Prevention (CDC) documented that the positive screening rate for anxiety among university students surged from 28.3% in 2019 to 41.6% in 2022 [10]. These findings highlight the urgent need to investigate effective strategies to mitigate anxiety among college students.

Based on Ulrich’s Stress Reduction Theory (SRT), some researchers proposed that natural environments can facilitate stress recovery by reducing physiological and psychological strain [11]. Similarly, Kaplan’s Attention Restoration Theory (ART) suggests that natural landscapes can restore people’s attention and cognitive functions [12]. Furthermore, the biophilia hypothesis, proposed by. Wilson in 1984, postulates that humans have an innate affinity for natural environments, which can evoke emotional pleasure, reduce anxiety, and enhance health [13]. Therefore, improving the quality of green spaces can effectively relieve the stress of adolescents [14–17], which has a significant impact on their physical and mental health and overall well-being. By addressing the negative aspects of urbanization through the integration of the natural elements into urban planning, we can create healthier living conditions. This approach not only benefits college students and adolescents but also contributes to the overall well-being of the urban population.

Studies have demonstrated that various landscapes types provide distinct benefits for physical and mental recovery. Both natural landscapes and artificial environments exhibit unique restorative effects. Natural landscapes such as forests, lakes and mountains have been shown to effectively alleviate stress, anxiety and even depression symptoms [18]. A recent study comparing fatigue recovery across four natural landscapes (grassland, public square, forest and lakeside) found that forest and lake had the strongest recovery effect in reducing negative emotions and enhancing positive emotions [18]. Artificial environments, including urban green spaces, parks and other leisure places, also play an important role in promoting physical and mental health. Jin et al. [19] reported that urban parks have a greater restoration effect than green streets. Wu et al. [20] emphasized that urban forest landscapes offer significant physical and psychological benefits for adolescents.

Although the quality of green spaces can be assessed by objective standards, their influence on health outcomes may depend on subjective perceptions and whether these attributes align with individual preferences and needs. A field experiment involving 485 participants exposed to real forest and urban landscapes found that 316 individuals experienced increased positive emotions while walking in the forest environment. However, 35% of the participants did not experience similar emotional changes [21]. This variation highlights the substantial individual differences in response to environmental stimuli, influenced by factors such as gender, age, cognitive ability and other personal characteristics. Gender, in particular, is a critical variable in psychological and behavioral studies [22–25]. Males and females often differ in their environmental perception, aesthetic preferences and behavioral responses. For instance, females tend to favor sheltered, quiet landscapes, whereas males generally prefer open and exploratory landscapes [23]. Age is another influential factor, as individuals at different developmental stages have varying restorative needs. Studies have shown that children have a strong dependence on nature, which declines during adolescence and stabilizes in early adulthood [26]. Moreover, age influences how individuals perceive audiovisual features in urban parks: younger prefer places with abundant visual elements, whereas elder pay more attention to the acoustic features of the scene [27]. Cognitive level also shapes environmental experience. Individuals from different professional backgrounds may exhibit distinct environmental needs and emotional responses [28]. Variations in disciplinary training and professional interests contribute to differences in landscape perception and preference, which in turn affect the perceived restorative potential of those environments [29]. Therefore, a nuanced understanding of how gender, academic level and disciplinary background impact individuals’ responses to landscapes is essential for further exploring the role of the environment in health recovery.

Previous studies have examined gender differences in stress recovery among university students [17,25,30], yet the influence of academic year and disciplinary background has received less attention. University enrollment represents a significant life transition, marking a critical period for social and mental health. Students in different grade level may encounter distinct challenges [31]. The freshman year is generally considered the least stressful year of university life [32], while the junior year is a period of emotional development conflicts [33], particularly marked by uncertainties regarding employment. The sources of stress vary significantly between these two years. Similarly, Beiter et al. [8] found that juniors scored higher than freshman on measures of depression, anxiety and stress. Based on the above, we selected freshmen and juniors as the grade level variables for analysis. Furthermore, research indicates that depression scores differ between male and female students at different stages of their undergraduate studies. Specifically, female students in their first and second years tend to have higher depression scores compared to their male counterparts. Conversely, studies suggest that male students in their third year exhibit higher depression scores than their female counterparts [34]. In East Asian societies, traditional cultural expectations place a dual burden on men, requiring them to fulfill both family responsibilities and broader social roles. Traditional cultural expectations require men to bear both family responsibilities and substantial social obligations. Correspondingly, gendered socialization patterns encourage emotional expressiveness in women (excluding anger), whereas men are typically socialized to inhibit emotional expression in favor of stoicism and self-restraint [35]. Therefore, a comprehensive analysis of differences in gender, grade level, and major among university students is crucial. It is necessary to deeply investigate individual differences among university students through this study to better elucidate the interaction between the environment and individuals and to explore how to maximize the restorative benefits of natural landscapes.

This study explores differences in how individuals across various grades, genders, and professions experience restorative environments to offer targeted recommendations for environmental design. Through quantitative surveys and psychological assessments, this study analyzes how different restorative environments affect diverse populations. By doing so, it aims to deepen understanding of how specific environmental characteristics influence the recovery processes of various groups. Specifically, the study is based on visual stimuli comprising pictures of different natural environments. It examines the changes and effects of spatial type, gender, grade level, and profession on adolescents’ physiological responses, emotional states, and preferences when exposed to natural environments. This study proposes the following hypotheses:

(H1) Females prefer and benefit more from sheltered landscapes like forests or artificial settings, while males favor and gain greater restoration from open, functional, and exploratory landscapes.

(H2) Juniors, facing higher academic stress, experience greater restorative effects than freshmen.

(H3) Landscape majors, with greater awareness of landscape elements, prefer and benefit more from complex, element-rich landscapes, while non-landscape majors favor modern, simple, and functional spaces.

Testing these hypotheses will provide critical insights into how restorative environments can be optimized to meet the specific needs of distinct demographic groups.

## 2. Materials and methods

### 2.1 Study participants

In this study, 105 participants were recruited through both online and offline advertisements; 7 invalid questionnaires were excluded, a total of 98 questionnaires were retained, comprising 48 males (49%) and 50 females (51%); 50 freshmen (51%) and 48 juniors (49%); and 58students majoring in landscape-related disciplines (59.1%, mainly involving the major of Landscape architecture) and 40 from non-landscape majors(40.9%, mainly involving the departments of Design and Chinese Language and Literature). All participants were undergraduate students from the University of South China, with a mean age of 19.6 ± 1.2 years (**Table 1**). The sample size is comparable to similar studies such as Samus et al. [36], Zhao et al. [37], and Yin et al. [38]. Eligibility criteria for participation were as follows: (1) being in good health with no history of physical or mental illness; (2) voluntarily signing an informed consent before the start of the experiment; and (3) agree not to smoke; drink alcohol, coffee or tea, or other potentially excitatory beverages the day before the experiment; and to refrain from strenuous physical activity within 24 hours prior to the study [14].

**Table 1:** Descriptive Statistics for Study Participants.

Categories	Gender	N	Percentage
Total	Male	48	49%
	Female	50	51%
Grade	Freshman	50	51%
	Junior	48	49%
Age	18	30	30.60%
	19	12	12.20%
	20	22	22.40%
	21	32	32.70%
	22	2	2%
Major (Planned Profession)	Landscape	58	59.10%
	Non-landscape	40	40.90%

All experimental procedures were conducted in accordance with the ethical standards of the National Research Committee and were approved by the Ethics Committee of the College of Architecture and Design, University of South China (prot. n. 18/2024).

### 2.2 Stimulus choice

Two-thirds of the human body’s perception of spatial stimuli originates from vision. Image studies are an economical and convenient research tool. It ensures that all participants receive identical visual stimuli, effectively eliminating the influence of uncontrollable environmental variables, such as weather, lighting, and crowd dynamics. Studies have found no significant differences between image-based research and natural stimuli [39,40]. This method has been widely applied in the fields of landscape and environmental research [41].

**Fig 1:**
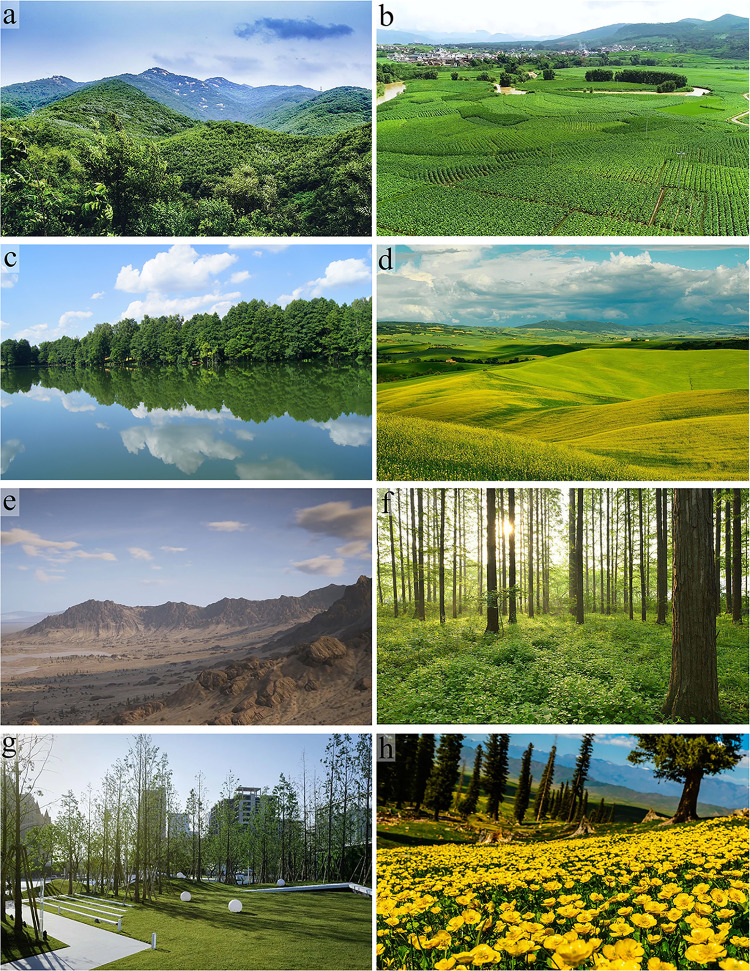
Eight images of experimental visual stimulation. a: mountain scenery; b: field; c: waterscape; d: lawn; e: desert; f: forest; g: artificial nature; h: plant.

Based on the analysis of landscape types identified in previous studies, this research collected high-resolution landscape photographs using the Bing search engine. The selected scenes were designed to replicate typical visual environments that participants might encounter in daily life, ensuring the real-world applicability of the experiment. Additionally, the diversity and representativeness of the scenes were key considerations. We aimed to include a variety of different environmental types to ensure the broad applicability of the study results while avoiding biases that might arise from overly restrictive scene selections. Eight typical landscape types were selected: mountains, fields, water-containing landscapes, grasslands, deserts, artificial nature, and floral-dominated plant landscapes. These images were cropped and uniformly resized to the same dimensions (**Fig 1**) to facilitate subsequent analysis. Through this approach, we aim to provide a systematic and diverse foundation of visual materials for the study.

### 2.3 Experimental design

The study experiment was conducted in a virtual simulation laboratory at the University of South China from March 27–30, 2024. The experiment was comprised three phases: preparation, induced stress, and recovery. Participants were randomly assigned to different conditions. For preparation, participants entered the classroom, and received materials and equipment. They filled out a basic socio-demographic questionnaire and were briefed on the experimental steps. Participants were then asked to sit quietly for five minutes to minimize external influences. To induce stress, participants engaged in an English language assessment beyond their typical proficiency, specifically above the Test for English Majors-Band 8 (TEM-8) level. The TEM-8 served as a calibrated stressor by leveraging the proficiency gap between non-majors (CET-4/6) and the pinnacle assessment for English majors. This intentional use of a more challenging language assessment was designed to induce acute stress. Previous studies have shown that non-native speakers experience anxiety when speaking a foreign language; as such, this approach replicated that stress [20]. Experimenters were trained to interact strictly with participants during this task and were instructed not to offer any comfort. Pre-test measurements of physiological indicators such as blood pressure and pulse were collected during this phase.

**Fig 2:**
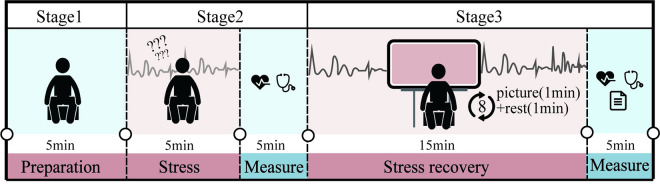
Fatigue recovery experimental process.

During the recovery phase, participants were divided into two groups. The control group sat quietly after the language test to observe changes in their physiological data. The experimental group (or image-viewing group) viewed eight prepared images. Each image was projected for one minute, followed by one minute of rest (**Fig 2**). During this period, participants were asked to fully experience the images in silence, without using electronic devices, talking, or eating. After participants viewed the images, post-test measurements of blood pressure and pulse were collected, along with measurements of Perceived Restorativeness Scale (PRS) scores and visual preferences ratings. To reduce interference, each participant viewed the images in a different order.

### 2.4 Measurements

#### 2.4.1 Physiological measurements.

Blood pressure and heart rate are key indicators of a physiological stress response, based on cardiovascular status [42]. When experiencing stressful situations, an individual’s heart rate becomes faster, breathing becomes deeper, and blood pressure rises. These cardiovascular system responses reveal a person’s psychological stress level in an objective and real-time way. Elevated levels of these indicators usually mean the person is in a state of agitation or anxiety [43]. Blood pressure includes systolic blood pressure (SBP) and diastolic blood pressure (DBP). Blood pressure and pulse rates were collected using a Cofoe electronic blood pressure monitor (KF-65C, Hunan, China). Blood pressure was measured using a Kufu blood pressure monitor (65C) before and after each image exposure, with readings taken one minute post-viewing. Participants were seated in a quiet, controlled environment, ensuring minimal external disturbances. They were positioned with supported backs, feet flat on the floor, and arms resting on a table, aligning the cuff with heart level. The cuff was securely placed 1–2 cm above the elbow, with the air tube directed over the brachial artery to ensure accuracy [44].

#### 2.4.2 Psychological stress.

The Perceived Restorativeness Scale (PRS), developed by Hartig (1997), is a widely used instrument for evaluating the restorative potential of environments [40]. It is based on Attention Restoration Theory to assess four key dimensions: “being away” (the extent to which the environment offers a mental escape from daily routines), “fascination” (the ability to effortlessly capture attention), “coherence” (the degree of perceptual and conceptual organization), and “compatibility” (the alignment between environmental features and an individual’s goals and preferences). This scale is expressed in declarative sentences and is measured using a 7-point Likert scale (1 = very unlikely, 7 = very likely) [45,46]. Participants filled out a modified version of the questionnaire several times during the experiment. To minimize excessive psychological burden and negative effects on participants, we used a shortened version of the PRS. Given the large number of images to be evaluated, it was impractical to use the full version. Therefore, we selected one representative item from each of the four dimensions (for a similar approach, see, e.g., Berto [47]; Felsten [48]; Herzog et al. [49]; Stragà et al. [50]).

#### 2.4.3 Visual Preference.

To assess how much participants liked each environment, participants reported their preferences for different landscape types using a five-point Likert scale (0 = “I don’t like it at all” 4 = “I like it a lot”).

### 2.5 Questionnaire design

The experiment assessed the level of attentional recovery for each spatial type using a questionnaire with three main sections: socio-demographic information, physiological responses (SBP, DBP and HR) after stress induction, and a perceptual restorability scale. The first section includes the subject’s personal background information, such as gender, college grade, age and major. The second part is the subject’s stress state after inducing stress. The third part is the PRS and its preference evaluation.

### 2.6 Data analysis

The statistical analysis was completed using the statistical software package SPSS 26.0 (IBM, Armonk, New York, USA). The change before and after viewing a picture is denoted by ∆ (∆ = post-picture value – pre-picture value). One-way analysis of variance (ANOVA) was used to analyze the significance of the physiological data of the control and experimental groups. An independent samples t-test was used to determine the mean and significance levels when comparing participants with genders and grades with respect to changes in their physiological data, before and after viewing the pictures. The t-test assumed a normal distribution. When the differences were significant, the post-hoc tests (LSD) method was used as a post hoc multiple comparison test to explore differences in variables between environments.

## 3. Results

### 3.1 Physiological response of viewing different landscape types

**Fig 3:**
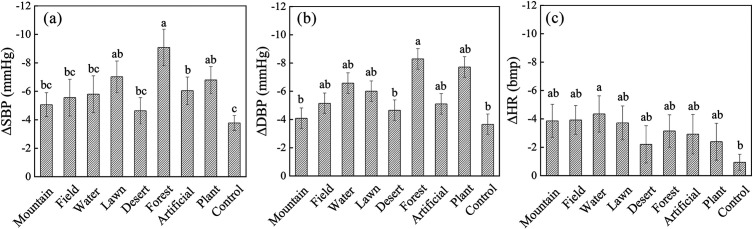
Comparation of changes in BP (blood pressure) and HR (heart rate) between the experimental (landscape viewing) group and control group when exposed to a stressor. ^a-c^post-hoc tests: means with the same superscript do not differ; SBP: systolic blood pressure, DBP: diastolic blood pressure, and HR: heart rate.

Participants exposed to specific images (∆forest = −9.08 ± 1.28, ∆lawn = −7.02 ± 1.10, ∆artificial = −6.04 ± 0.96, ∆plant = −6.79 ± 0.94) showed significantly greater reduction in systolic blood pressure (all p < 0.05, *η*^2^ (Partial) = 0.03, **Fig 3a**) compared to the control group (∆control = −3.71 ± 0.52). Forest images also elicited a more pronounced decrease (t = −2.68, p = 0.009) in diastolic blood pressure (∆forest = −8.28 ± 1.30) compared to the control (∆ control = −3.65 ± 1.17) (**Fig 3b**). Furthermore, heart rate declined more sharply (t = −2.44, p = 0.017) after viewing waterscapes (∆water = −4.34 ± 1.27) were greater than in control group (∆ control = −0.93 ± 0.56).

#### 3.1.1 Differences in gender, grade level and major.

**Fig 4:**
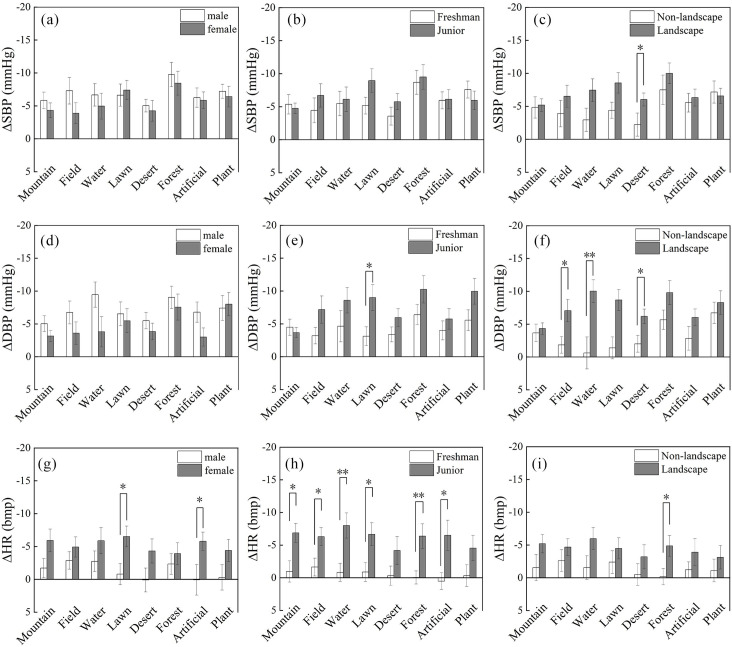
Differences in physiological changes of participants from exposure to different landscape spaces. SBP: Systolic blood pressure; DBP: diastolic blood pressure; HR: heart rate.

Systolic blood pressure. No significant differences were observed between gender or grade level groups (Fig 4a, 4b). When viewing desert landscape images, landscape majors showed a greater decrease in ΔSBP (−6.03 ± 0.97 mmHg) compared to non-landscape majors (−2.22 ± 1.74 mmHg; t = −2.06, p = 0.045, Cohen’s d = 0.61; Fig 4c). However, when viewing other landscape images, no significant differences in ΔSBP were observed between the two majors.

Diastolic blood pressure. Among the eight types of spatial landscapes, no significant differences in ΔDBP were observed between males and females (Fig 4d). When exposed to the lawn environment, ∆DBP was significantly lower in junior year participants (−9.00 ± 1.97 mmHg) compared to freshman year participants (−3.12 ± 1.43 mmHg) (t = −2.42, p = 0.02, Cohen’s d = 0.69; Fig 4e). There were significant differences in ∆DBP between landscape and non-landscape majors when viewing pictures of fields, waterscapes, and deserts. For the field picture, ∆DBP (−7.06 ± 1.70) was significantly lower for landscape majors than in non-landscape majors (−1.83 ± 1.26) (t = −2.13, p = 0.03, Cohen’s d = 0.63, Fig 4f). For the waterscape picture, ∆DBP was significantly lower for participants in landscape majors (−10.23 ± 1.73) compared to non-landscape majors (−0.61 ± 2.4) (t = −3.20, p = 0.002, Cohen’s d = 0.95). For the desert landscape, ∆DBP was significantly lower for participants in landscape majors (−6.19 ± 1.10) than for non-landscape majors (−2.00 ± 1.28) (t = −2.39, p = 0.02, Cohen’s d = 0.71).

Overall, gender did not show significant differences in diastolic blood pressure (DBP) restoration. Students in higher grade level demonstrate superior DBP restoration in lawn environments. Students majoring in landscape show more pronounced DBP physiological restoration effects in grassland, water, and desert settings.

Heart rate. In the lawn environment, females (−6.52 ± 1.58) exhibited significantly higher heart rate changes (∆HR) compared to males (−0.79 ± 1.62) (t = 2.53, p = 0.02, Cohen’s d = 0.72). A significant difference in ∆HR between males and females was observed when viewing pictures of lawn and artificial settings (Fig 4g). Significant differences in heart rate recovery between grade levels were observed among participants viewing pictures of mountain landscapes, fields, waterscape, lawns, forests, and artificial landscapes (Fig 4h). For the mountain picture, ∆HR was significantly lower for juniors (−6.87 ± 1.45) than for freshmen (−0.96 ± 1.62) (t = 2.69, p = 0.01, Cohen’s d = 0.77). After viewing the field picture, juniors (M = −6.29 ± 1.38) experienced a significantly lower ∆HR than freshman (M = −1.64 ± 1.36) (t = 2.39, p = 0.02; Cohen’s d = 0.68). For the lawn picture, ∆HR was significantly better for juniors (M = −6.67 ± 1.75) than for freshmen (−0.88 ± 1.44) (t = 2.55, p = 0.01, Cohen’s d = 0.72). For the waterscape picture, ∆HR was significantly lower for juniors (−8.0 ± 1.93) than for freshmen (−0.88 ± 1.44) (t = 3.03, p = 0.004, Cohen’s d = 0.86). For the forest picture, ∆HR was significantly lower for juniors (−6.37 ± 1.88) than freshmen year (−0.04 ± 1.00) (t = 2.97, p = 0.005, Cohen’s d = 0.85). For the artificial picture, the ∆HR for junior landscape majors (−6.50 ± 2.31) was significantly lower compared to freshman landscape majors (0.52 ± 1.28) (t = 2.67, p = 0.01, Cohen’s d = 0.76) (Fig 4i). Additionally, ∆HR differed significantly between majors for the forest picture, with landscape majors (M = −4.87 ± 1.57) showing a significantly lower ∆HR than non-landscape majors (M = 0.16 ± 1.25) (t = −2.33, p = 0.02, Cohen’s d = 0.61).

Overall, females exhibit superior HR restoration in field landscapes. Juniors demonstrate significantly better HR restoration across various landscapes compared to freshman. Students majoring in landscape show enhanced heart rate restoration in forest and artificial natural landscapes. Gender, grade level, and professional background significantly influence participants’ heart rate restoration across different landscape types, with students in landscape architecture and higher grade level exhibiting stronger physiological restoration capabilities.

### 3.2 Psychological response of viewing different landscape types

The eight landscape types differed significantly in promoting psychological recovery among participants. Additionally, there were significant differences between individuals with respect to the impact of different landscape types.

**Fig 5:**
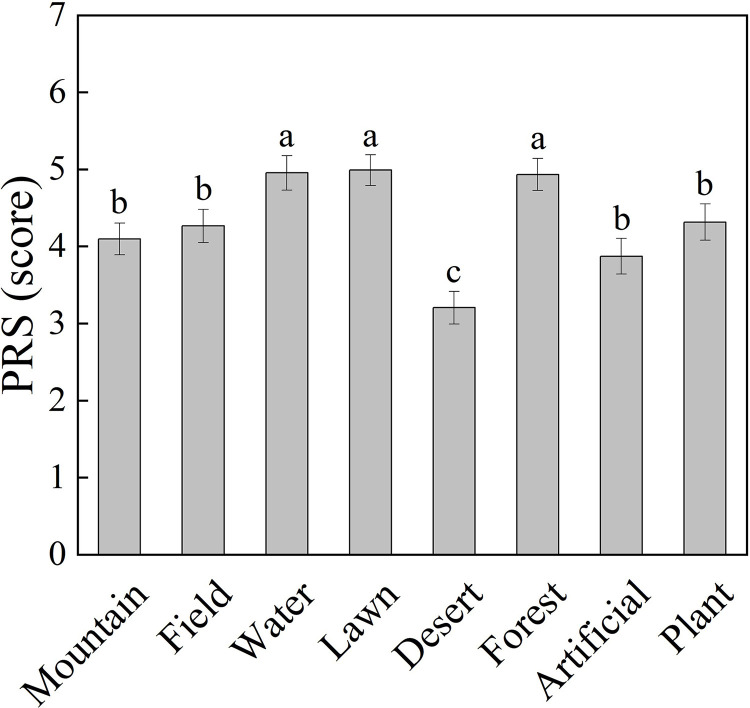
Comparisons of participants’ mean ratings of the landscape spatial perception restoration scale. ^a-c^post-hoc tests: means with the same superscript do not differ.

The internal reliability of the picture restorability evaluation scale was analyzed with a significance level of p < 0.05 and an alpha coefficient of 0.833, indicating high data reliability. The results of the one-way ANOVA indicated a significant difference in the total scores representing the eight spatial restorability scores (F = 8.275, p < 0.05, *η*^2^ (Partial) = 0.13). There was significant variation in the level of restoration among different spatial types. The overall scores indicated the restorative capacity provided by the 8 spatial types was highest for grass space, followed in descending order by water landscape, forest space, plants, field space, mountain space, artificial nature, and desert space. Lawn space had the highest restorative effect with respect to stress recovery, while desert space had the lowest restorative effect (**Table 2**). The one-way analysis showed that grassland, waterscape, and forest were associated with significantly higher scores than the remaining five landscape spaces; the desert was associated with significantly lower scores than the other seven landscape pictures (**Fig 5**).

**Table 2:** Mean Score (mean ± standard error) of Perceived Restorative Scale.

Group	Mountain	Field	Water	Lawn	Desert	Forest	Artificial	Plant
Total	4.09 ± 0.20	4.26 ± 0.21	4.95 ± 0.22	4.98 ± 0.19	3.20 ± 0.21	4.93 ± 0.20	3.87 ± 0.23	4.31 ± 0.23
Male	4.05 ± 0.27	4.52 ± 0.28	4.93 ± 0.26	4.87 ± 0.20	3.34 ± 0.35	4.86 ± 0.29	3.95 ± 0.36	4.06 ± 0.33
Female	4.14 ± 0.30	4.02 ± 0.32	4.97 ± 0.36	5.10 ± 0.34	3.07 ± 0.24	5.0 ± 0.29	3.79 ± 0.29	4.56 ± 0.32
Freshman	3.75 ± 0.30	3.79 ± 0.34	4.53 ± 0.34	4.51 ± 0.29	3.13 ± 0.33	4.68 ± 0.32	3.18 ± 0.31	3.80 ± 0.33
Junior	4.45 ± 0.25	4.76 ± 0.22	5.39 ± 0.26	5.48 ± 0.22	3.28 ± 0.27	5.19 ± 0.25	4.59 ± 0.28	4.85 ± 0.30
Landscape major	4.48 ± 0.23	4.70 ± 0.22	5.29 ± 0.24	5.31 ± 0.19	3.45 ± 0.25	5.13 ± 0.24	4.54 ± 0.26	4.76 ± 0.26
Non-landscape major	3.43 ± 1.38	3.5 ± 0.32	4.43 ± 0.42	4.43 ± 0.40	2.77 ± 0.37	4.58 ± 0.36	2.70 ± 0.27	3.54 ± 0.39

**Fig 6:**
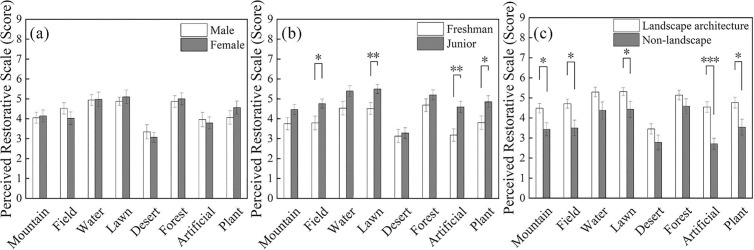
Illustrates the differences in participant ratings on the perceived restoration scale across forest landscape spaces, including gender differences, grade level differences, and academic major differences (N = 49; Mean ± SE; * p < 0.05, ** p < 0.01, *** p < 0.001).

The results indicated no significant differences in restorative ratings between male and female; however, there were significant differences with respect to grade level and major (**Fig 6**). There were no significant differences in participants’ restorative scores between males and females. Males exhibited the highest restorative scores in waterscape scenes (4.93 ± 0.26), while females showed the highest scores in forest scenes (5.0 ± 0.29). Both genders recorded the lowest restorative scores in desert scenes (males: 3.34 ± 0.35; females: 3.07 ± 0.24; **Fig 6a**). Junior-year participants reported significantly higher restorative ratings than freshman participants for the field setting (t = −2.34, p = 0.023, Cohen’s d = 0.66), lawn setting (t = −2.59, p = 0.013, Cohen’s d = 0.74), Artificial environment (t = −3.35, p = 0.002, Cohen’s d = 0.95), and plant environment (t = −2.33, p = 0.024, Cohen’s d = 0.66). No significant differences were observed in restorative ratings for the mountain, water, desert, or forest scenes; however, junior participants’ evaluations were generally higher than those of freshman participants (**Fig 6b**). Restorative ratings from landscape majors were significantly higher than those from non-landscape majors for the mountain scene (t = 2.63, p = 0.011, Cohen’s d = 0.74), field setting (t = 2.89, p = 0.006, Cohen’s d = 0.85), lawn setting (t = 2.22, p = 0.031, Cohen’s d = 0.65), artificial environment (t = 4.53, p < 0.0001, Cohen’s d = 0.95), and vegetated environment (t = 2.65, p = 0.011, Cohen’s d = 1.34) (**Fig 6c**).

Overall, males and females exhibit highly similar psychological restoration scores. Third-year students demonstrate higher restoration scores compared to first-year students. Students majoring in landscape architecture show higher restoration scores compared to those in non-landscape-related majors.

### 3.3 Visual preference analysis

Overall, the highest preference score was for the waterscape space (3.18 ± 0.15), which is consistent with the strong impact of urban water bodies on environmental restoration noted in previous studies [51]. The lowest preference score was associated with the desert picture (1.86 ± 0.16).

**Fig 7:**
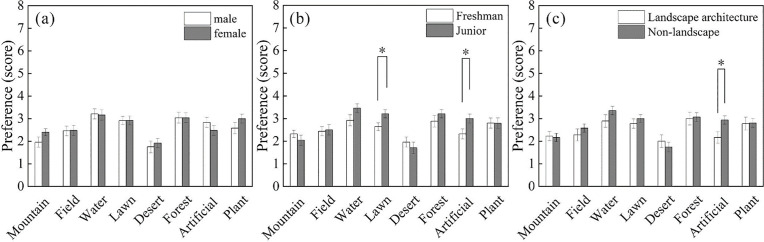
Illustrates the difference in preference scores among participants exposed to different types of landscape spaces (N = 49; Mean ± SE; * p < 0.05, ** p < 0.01, *** p < 0.001).

Preference scores did not differ significantly by gender but showed significant differences across certain grade levels and majors (**Fig 7**). Specifically, participants in different grade levels reported different preferences for the lawn environment (t = −2.28, p = 0.027, Cohen’s d = 0.65), with juniors preferring the lawn space more than freshmen. Similarly, participants in different grade levels reported different preferences for the artificial environment (t = −2.27, p = 0.028, Cohen’s d = 0.65), with juniors preferring the artificial environment more than freshmen. In addition, participants in different majors reported significant difference in the preferences for artificial environment (t = 2.50, p = 0.016, Cohen’s d = 0.74). Landscape majors had a higher preference for the artificial environment than non-landscape majors.

## 4. Discussion

This study investigates the restorative effects of diverse landscape spatial characteristics on college students, focusing on the interactive influences of gender, grade level, and major on physiological and psychological restoration in various natural environments. While previous research has demonstrated that natural environments exert greater restorative benefits compared to urban settings, few studies have concurrently examined the roles of grade level and major alongside gender. Using picture-based simulation experiments, we tested three hypotheses: (H1) Females prefer and benefit more from sheltered landscapes like forests or artificial settings, while males favor and gain greater restoration from open, functional, and exploratory landscapes.; (H2) Juniors, facing higher academic stress, experience greater restorative effects than freshmen; and (H3) landscape majors, with greater awareness of landscape elements, prefer and benefit more from complex, element-rich landscapes, while non-landscape majors favor modern, simple, and functional spaces. However, our findings showed that in terms of physiological restoration effects, males exhibited better restoration in lawn settings, whereas the differences in Perceived Restorativeness Scale (PRS) and preference scores between genders were negligible. Consistently, both physiological and psychological indicators provided substantial support for the hypothesis that third-year students experienced better restoration effects than first-year students. Additionally, there was partial support for hypothesis H3, with students in landscape-related majors showing better restoration effects compared to those in non-landscape majors.

### 4.1 Restoration effects vary across landscapes

This study addresses the limitations of prior research regarding the impacts of different environmental types by systematically evaluating eight representative landscape types. The results indicated that participants experienced varying degrees of psychophysiological recovery after observing eight different types of landscapes. Results obtained for subjects viewing different landscape types indicated differences in their recorded SBP, DBP, HR, and PRS scores. These findings are consistent with previous research finding that different feature spaces have different effects on cardiovascular relaxation, psychological well-being, and recovery from perceived stress [52,53]. Of the eight landscape spaces, water features and forests were the most restorative. Several studies have found forests to be more relaxing than other restorative environments [54] and may be better suited as restorative environments for relaxation [19]. Water feature spaces also have high restorative effects, consistent with previous findings that natural environments containing water produce higher perceptions of restoration than natural scenes without water [55], and that water promotes restorative evaluations of environments covered in green plants [56].

### 4.2 Differential sensitivity of populations to landscape

#### 4.2.1 Juniors exhibit faster recovery and more positive preference than freshmen.

Among the eight landscape types, junior students experienced a higher restoration effect than freshmen, as indicated by physiological data, after exposure to six landscape types (mountain, field, waterscape, lawn, forest, and artificial nature). However, juniors showed better physiological indicators of recovery compared to freshmen. In terms of psychological indicators, landscapes such as fields, lawns, artificial nature, and plant settings were associated with significantly higher recovery scores for juniors than freshmen. While no significant differences were found for exposure to mountain, water, desert, and forested landscapes, the recovery scores were generally higher for juniors than for freshmen.

This difference in recovery between grade levels can be partially attributed to age. Other studies on age and stress have shown that adults typically have a more negative stress mindset compared to young adults (mid- and early-career). Older adults have lower cortisol and heart rate responses to stressful stimuli, and they report fewer subjective stress and negative stress appraisals than younger adults [56]. However, older individuals experience a slower heart rate recovery in response to unpleasant emotions.

Different from previous studies, this research found college juniors tend to exhibit greater psychological and emotional maturity than freshmen. They have gathered richer learning and life experiences and are more competent in managing and adapting to stress. As they progress through their college years, students begin to form ideas and improve their overall comprehensive abilities, including their ability to cope with stress. Due to their advanced psychological maturity and self-regulation skills, junior-level students are better equipped to take advantage of the restorative benefits of natural environments. Furthermore, they may also have a greater appreciation for nature’s beauty.

Individuals with a strong connection to nature are more likely to appreciate its restorative power, enhancing the positive effects of natural environments on their well-being. By their junior year, students usually establish a more stable and supportive social network consisting of friends, classmates, and mentors. Spending time with friends in natural settings may be an effective form of stress relief, as companionship provides a sense of safety and contributes to recovery. Overall, these factors work together to enable individuals to maximize the restorative potential of natural environments, resulting in better psychological and physical recovery. These findings have important implications for educational practice and provide useful references for promoting students’ mental and physical health. These findings have important implications for educational and mental health practitioners. The differences observed between freshman and junior students suggest that developmental stage and social context influence restorative experiences. Practitioners can apply these insights by designing nature-based well-being programs for different educational stages. For example, high school students could benefit from structured outdoor activities that foster nature connectedness and social bonds, while college students may find stress relief in peer-based green space activities. Extending these findings to both high school and university settings can help tailor interventions to better support students’ mental and physical health.

#### 4.2.2 The effects of landscape type on fatigue recovery various among students with different major.

This study found that landscape majors significantly outperformed non-landscape majors in physical and psychological recovery across multiple landscape types. Further analyses showed that the effect of academic major was very pronounced. Among the eight landscape types, the restoration effect was higher for landscape majors than non-landscape majors, as measured by physiological data, after exposure to four landscape types (field, waterscape, forest, and desert). In terms of subjective ratings, landscape majors reported a significantly better recovery than non-landscape majors when exposed to four landscape types (mountain scenes, field scenes, lawns, artificial nature, and plants). In terms of landscape preference, landscape majors had a significantly higher preference for artificial nature than non-landscape majors. Although there were no significant differences in the subjective restoration scores for desert (as discussed further below), mountain, water, forest, and plantation landscapes among different academic majors, landscape majors reported higher scores compared to non-landscape majors.

Landscape majors’ higher familiarity with different landscape types and their specialized educational backgrounds may explain these outcomes. Familiarity and experience play a critical role in individuals’ evaluations of the emotional benefits of natural environments [56–59]. Research indicates a positive correlation between landscape familiarity and preference [60], and life experiences and familiarity also play significant roles in the health benefits derived from natural environments [38]. For instance, residents living in desert areas exhibit significantly higher preference scores for desert landscapes compared to non-residents [61]. This finding aligns with the generalized insecurity theory of stress [56,62], which posits that as familiarity with an environment increases, perceived insecurity factors decrease, thereby facilitating stress recovery. Familiar environments enable individuals to more rapidly identify safety cues. Furthermore, individuals’ restorative experiences are closely related to their personal characteristics [63] and educational background [64]. Previous studies have shown that participants from different professional backgrounds exhibit significant differences in image evaluations, particularly among students majoring in landscape architecture, horticulture, and agronomy, who provide higher restorativeness and preference ratings [65]. Similar studies have found that a survey analysis of 205 Canadian students revealed that those majoring in outdoor recreation and tourism demonstrated significantly higher environmental awareness and ecological cooperation compared to students in other majors. This suggests that students in environmental disciplines tend to have stronger ecological awareness and behavioral intentions [66]. We hypothesize that this phenomenon is closely linked to the knowledge advantages of landscape architecture majors, particularly in ecological cognition and understanding of natural environments. Landscape architecture professionals typically possess strong ecological cognitive abilities, which enable them to better adapt to and relax in natural environments, thereby promoting physiological recovery. Shatalova (2023) noted that when studying the impact of human-nature interactions on restorative effects, “top-down” variables (such as personality traits, values, and emotional connections to nature) should be considered. Specifically, individuals with higher nature connectedness and environmental values are more likely to derive health benefits from nature experiences [67]. From this theoretical perspective, students in environment-related design disciplines, due to their educational background and interest inclinations, typically exhibit deeper emotional connections to nature and stronger ecological cognition. Consequently, their psychological restorative effects from nature exposure may be more pronounced. These findings suggest that educators and mental health professionals can enhance stress reduction programs by leveraging students’ deeper connection to nature, especially in environment-related disciplines. Nature-based activities could be incorporated to maximize psychological benefits. Additionally, campus outdoor spaces can be designed to align with students’ ecological values to further promote restorative effects.

### 4.3 Partial landscape specificity

Desert landscapes did not significantly affect grade level or gender in terms of overall restoration effectiveness. However, there were significant differences in physiological responses among academic major groups. We believe this result may be related to the unique and extreme nature of desert environments. Throughout evolutionary history, people have preferred landscape features that support their survival [56]. Deserts typically evoke perceptions of resource scarcity and danger [68], leading to generally lower preferences and physiological responses [69] because deserts represent a scarcity of water, food, and habitat. Han’s (2007) study also indicated that, compared to other green or water-containing landscapes, desert landscapes received the lowest preference [70]. Humans may prefer cues indicating the presence of food and resources with high survival value. There is substantial evidence that humans prefer open, expansive savannas with low, uniform vegetation and clear water, preferences that imply deep mechanisms of evolutionary psychology [71]. However, an individual’s perception of landscapes is shaped not only by genetic factors but also cultivated throughout their lifetime. Li et al. (2022) posit that millions of people raised in desert environments worldwide may find these ecosystems conventional and familiar [72]. Consequently, deserts can function as supportive environments for local inhabitants. Supportive Environment Theory (SET) helps explain how natural landscapes can improve health. SET suggests that people need easy-to-understand and manageable environments to maintain their physical and mental well-being [73]. For students in landscape-related majors, professional education may enhance their exposure to and understanding of different natural environments, especially extreme ones like deserts. Their professional background makes them more familiar with desert environments than students in non-landscape-related majors. Although deserts may be unfavourable for survival from an evolutionary psychology perspective, these students, with their deeper understanding of ecosystems, may focus more on the unique ecosystems and biological adaptations in deserts rather than merely viewing them as resource-poor and environmentally harsh. Their experience in biology and ecology leads to a different perception of desert environments, where innate programmed responses may be altered by acquired specific environmental experiences, thus tending to prefer familiar biomes [71,74]. Research indicates that biodiversity is positively correlated with landscape aesthetic evaluations, but perceived biodiversity varies depending on the respondent’s educational background and professional experience [7]. Therefore, students in landscape-related majors may more readily find opportunities for restoration and relaxation in environments like deserts, consistent with previous research. Frequent exposure to and familiarity with specific natural environments, even those without direct nutritional value, may enable individuals to transcend the predictions of evolutionary psychology regarding the restorative benefits of savannas and other green natural elements. This phenomenon also aligns with the generalized insecurity theory, which posits that familiar environments aid humans in assessing safety cues [38]. These findings suggest that students in landscape-related majors may have different responses to desert landscapes compared to other disciplines. Educators and practitioners can use this insight to design nature-based programs that account for students’ familiarity with diverse environments. Exposure to various landscapes, including extreme ones like deserts, can enhance restorative experiences and well-being for students with such backgrounds.

### 4.4 Limitations

This study has some limitations. First, the subject population was limited to freshmen and junior students with specific majors at a specific university. Future studies should expand the sample to include liberal arts and science students, and students at other grade levels, to explore the impact of different educational backgrounds on restoration effects. In addition, It should investigates how cultural background and personalities impact landscape recovery effects.

Second, multisensory approaches are important in designing healthy environments [75,76]. Virtual and simulated natural landscapes have a positive impact but are often less effective than real landscapes [70,77]. Pictures provide visual stimuli but do not encompass sound and odor experiences. Future studies should combine auditory, olfactory, and tactile stimuli, or explore environmental restoration features in urban parks and use wearable devices to assess restoration effects. Furthermore, it is important to note that the landscapes provided in this study may differ in terms of openness. Open and semi-open spaces have been shown to play a significant role in participants’ ratings of natural environments in previous studies [37,78]. This factor should be considered as a limitation and future studies should account for variations in landscape openness when evaluating restoration effects. Finally, studies should be extended to include more scenarios, rather than being limited to landscape environments.

A specific limitation of the experimental design is the absence of a dedicated baseline physiological measurement prior to the stress manipulation phase. Physiological data were collected only immediately before and after image exposure. While this approach aligns with methodologies used in related studies to assess restorative effects (Wu et al. [20]; Liu et al. [35]), we acknowledge that establishing a true pre-manipulation baseline would enhance the robustness and reliability of our findings. Consequently, future research should incorporate baseline physiological data collection to improve methodological rigor. This will enable a more accurate assessment of environmental stimulus impacts and strengthen the validity of conclusions.

## 5. Conclusion

Many studies have verified the beneficial effects of the natural environment on human health. However, this study is unique because it systematically analyzes student grade level and significant factors in stress recovery, in addition to gender. The findings indicate that students at the higher grade level may be more adept at utilizing their surroundings to alleviate stress compared to those at the lower grade level. Students majoring in landscape architecture show higher physical and mental recovery levels compared to those in non-landscape majors. This academic training may lead people with inherent cognitive differences to exceed individual expectations. For example, the psychological needs and emotional states of participants exposed to desert landscapes vary greatly with their personality, experience, and values. This means there is no one straightforward way to meet everyone’s psychological demands. When evaluating the natural environment, it is important to consider both physiological and psychological factors and balance them to achieve the optimal recovery effect. These conclusions add to the field of environmental psychology and provide recommendations that can inform educational practice and environmental design. Future research should explore specific recovery mechanisms in different settings and apply this knowledge to optimize student learning and living environments.
